# Cardiac Monitoring and Heart Failure in Advanced Breast Cancer Patients Treated With Trastuzumab in Ontario, Canada

**DOI:** 10.3389/fcvm.2022.850674

**Published:** 2022-05-19

**Authors:** Moira Rushton, Coralea Kappel, Isac Lima, Meltem Tuna, Kathleen Pritchard, Steven Hawken, Susan Dent

**Affiliations:** ^1^The Ottawa Hospital Cancer Centre, University of Ottawa, Ottawa, ON, Canada; ^2^Department of Medicine, McMaster University, Hamilton, ON, Canada; ^3^The Ottawa Hospital Research Institute, Institute for Clinical Evaluative Sciences (ICES) UOttawa, University of Ottawa, Ottawa, ON, Canada; ^4^Sunnybrook Odette Cancer Centre, Toronto, ON, Canada; ^5^Duke Cancer Institute, Duke University, Durham, NC, United States

**Keywords:** breast cancer, cardio-oncology, cardiotoxicity, trastuzumab, heart failure

## Abstract

**Background:**

Trastuzumab has improved patient outcomes in HER2 + breast cancer (BC) but carries a risk of cardiotoxicity. Routine cardiac imaging is recommended for advanced breast cancer (aBC) patients during trastuzumab treatment despite a lack of evidence that this improves patient outcomes. This study was conducted to understand predictive factors for cardiac events and determine the impact of cardiovascular monitoring in aBC.

**Methods:**

This retrospective population-based cohort study included aBC patients treated with trastuzumab (all lines), in Ontario, Canada from 2007 to 2017. The overall cohort was divided into two groups; those who developed a cardiac event (CE) vs. those who did not. Patients with pre-existing heart disease were excluded. Logistic regression was performed to identify patient characteristics associated with an increased risk of CE.

**Results:**

Of 2,284 patients with HER2 + aBC treated with trastuzumab, 167 (7.3%) developed a CE. Median age at first dose of trastuzumab was 57 (IQR 49–66); 61 (IQR 51–70) for patients with a CE. Median number of cycles was 16 (IQR 7–32); 21 (IQR 8–45) for patients with a CE (*p* < 0.01). Twelve (0.5%) patients died of cardiac causes; all had a prior CE. Increased risk of CEs was associated with age > 60 (OR 5.21, 95% CI 1.83–14.84, *p* = 0.05) and higher number cycles of trastuzumab (OR 1.01; 95% CI 1–101, *p* = 0.028).

**Conclusion:**

This is the first population-based study to report on CEs and cardiac monitoring in HER2 + aBC patients during trastuzumab-based therapy. Older age and longer treatment with trastuzumab were associated with an increased risk of a CE.

## Introduction

Trastuzumab, a monoclonal antibody against human epidermal growth factor 2 (HER2) is the cornerstone of modern therapy for HER2 positive (+) advanced breast cancer (aBC). HER2 is overexpressed in approximately 15% of breast cancers (1–3) and therapies that target this pathway have significantly improved breast cancer outcomes for this population. Trastuzumab is generally a well-tolerated drug but comes with a risk of cardiotoxicity (4, 5). Trastuzumab induced-cardiotoxicity (TIC) presents as either asymptomatic drops in left ventricular ejection fraction (LVEF) or overt heart failure (HF) (6–8). The initial observation of TIC led to the inclusion of routine cardiac monitoring in all subsequent clinical trials with anti-HER2 targeted therapies, as well as international consensus guidelines and FDA recommendation for routine cardiac monitoring every 3 months in all patients treated with trastuzumab-based therapy (9–11).

The risk of trastuzumab induced cardiotoxicity has been well-studied in the adjuvant setting. A meta-analysis of adjuvant trastuzumab trials found a 2.5% incidence of heart failure (HF) in trastuzumab treated patients vs. 0.4% in those treated with chemotherapy alone (RR 5.11 90% CI 3.00–8.72) (12, 13). The 2014 Cochrane review reported that 4.7% of patients with aBC had a severe cardiac event vs. 1.1% in the chemotherapy arm (RR 3.49, 95% CI 1.88–6.47, *p* < 0.001) (14). The use of sequential anti-HER2 targeted therapies has led to patients living longer with HER2 + aBC (15). In the end-of-study analysis of the CLEOPATRA trial, patients in the experimental arm (trastuzumab, pertuzumab, docetaxel) received a median of 24 cycles with a mean overall survival of 57.1 months (95% CI 50–72) and the 8-year landmark analysis reported an overall survival of 37% (31–42) (16). Only 4.4% of patients were found to meet criteria for cardiotoxicity in the dual anti-HER2 arm and 1.2% experienced symptomatic drops in LVEF (17).

While well-intentioned, the high frequency of cardiac monitoring recommended by the FDA in this patient population has led to an increased detection of asymptomatic drops in LVEF (18), the clinical significance of which is unknown. The American Society of Clinical Oncology has made a moderate strength recommendation based on low-quality evidence to perform routine echocardiographic surveillance indefinitely in patients with aBC who are receiving trastuzumab with the frequency determined by healthcare providers (11). However, population-based studies have reported that detection of early (asymptomatic) cardiotoxicity in the adjuvant setting places patients at risk of not completing their intended anti-HER2 targeted therapy, and thus increasing their risk of cancer recurrence and death (19). In Ontario, Canada, cancer care is provided through a single-payer health care system which requires LVEF monitoring in aBC HER2 + patients on anti-HER2 targeted therapy every 3–6 months (20). The objective of this study was to examine patients with HER2 + aBC to determine the incidence of cardiac events and cardiac death as well as examine clinical factors that may impact risk of cardiac complications.

## Materials and Methods

### Study Design

This is a retrospective population-based cohort study of adult aBC patients (age > 18) treated with trastuzumab-based therapy in Ontario, Canada between January 1, 2007 and December 31, 2017. Patients with a history of heart failure or cardiomyopathy were excluded. The main exposure was treatment with trastuzumab given for palliative intent in any treatment line. In this provincial database with a single-payer system, access to anti-HER2 targeted therapies is only available to patients who overexpress HER2, therefore this was a surrogate for the presence of HER2 + status. Cases were defined by development of a cardiac event (CE). A CE was defined as new onset heart failure, pulmonary edema, or cardiomyopathy between initiation of trastuzumab and 90 days after the last dose, or death from any cardiovascular cause. The cohort was divided into two groups for comparison: group A who had a CE vs. group B who had no CEs during the study period. Ethics approval was obtained from the Ottawa Health Sciences Research Ethics Board, Ottawa, Canada.

### Endpoints

The primary outcome of this study was the incidence of a CE in aBC patients treated with trastuzumab-based therapy. The secondary outcomes were to assess the incidence of cardiac death and to examine clinical factors associated with increased odds of cardiac events in this population.

### Data Sources

Patients were identified using health administrative databases in Ontario, Canada that contain patient-level information on cancer diagnosis, cancer drug administration, inpatient and outpatient data, cancer registry data, and demographics. De-identified databases were accessed through the Institute for Clinical and Evaluative Sciences (ICES), and all data sources were linked through a unique encrypted identifier and analyzed at ICES. ICES is an independent, non-profit research institute funded by an annual grant from the Ontario Ministry of Health and Long-Term Care. As a prescribed entity under Ontario’s privacy legislation, ICES is authorized to collect and use health care data for the purposes of health system analysis, evaluation, and decision support. Secure access to this data is governed by policies and procedures that are approved by the Information and Privacy Commissioner of Ontario.

The Ontario Health Insurance Plan (OHIP) is a publicly funded provincial health insurance plan that covers medical costs for all residents in Ontario. The OHIP database was used to identify outpatient visits, physician visits, echocardiogram, and multigated acquisition scan (MUGA) testing through billing codes in our patients. The Ontario Cancer Registry (OCR) was used to confirm breast cancer diagnosis, cancer staging, estrogen receptor (ER) status, and progesterone receptor (PR) status information. HER2 status was not available but implied based on treatment with trastuzumab. Patients treated with trastuzumab were identified through the New Drug Funding Program (NDFP), a public health program which captures information on trastuzumab administration. The Canadian Institute for Health Information (CIHI) Discharge Abstract Database (DAD), CIHI same-day surgery, the National Ambulatory Care Reporting System (NACRS) database, and ICES-derived cohorts for pre-treatment Charlson co-morbidity index scores, treatment location (community vs. teaching hospital), ambulatory and emergency department visits and hospitalizations for heart failure, pulmonary edema and cardiomyopathy, income demographics, and cause of death were collected. Chemotherapy information was accessed through the cancer activity level reporting (ALR) database. We did not have access to LVEF values or clinic notes for this study.

Data collected included: age at diagnosis, *de novo* vs. recurrent disease, ER/PR status, history of anthracycline treatment, number cycles of trastuzumab, frequency of cardiac imaging, concurrent pertuzumab use, year of treatment, cause of death, CEs, Charlson co-morbidity index, and community vs. academic hospital setting. Cardiology visits were included in the descriptive analysis but not as a variable in the logistic regression model.

### Statistical Methods

Descriptive statistics were performed to report the baseline characteristics of patients in each group [CE (or group A) vs. no CE (or group B)]. Categorical variables were compared using a chi-square test or a Fisher exact test. Means for continuous variables were compared using Student’s *t*-test. Statistical significance was tested at an alpha of 0.05.

Logistic regression was carried out on the two groups to calculated odds ratios (OR) for factors predictive of CEs in this population. Patients who developed a CE (group A) were compared to patients who did not develop a CE (group B). Results are presented as odds ratios (ORs) with 95% confidence intervals (CI). The analyses were performed with SAS Software (version 9.4.3.0).

## Results

There were 2,284 patients with HER2 + aBC included in this study. Overall, the median age at first treatment was 57.0 (IQR 49.0–66.0) and patients received a median of 16.0 cycles of trastuzumab every 3 weeks (IQR 7.0–32.0). In our cohort, 25% (*n* = 572) of patients received concurrent pertuzumab therapy for aBC. Similarly, 25.9% (*n* = 592) had received anthracyclines in the previous 10 years and only 5.2% (*n* = 118) had received previous trastuzumab. The majority of patients in this study (62.6%, *n* = 1430) had stage IV disease (*de novo*) at the time of diagnosis.

**FIGURE 1:**
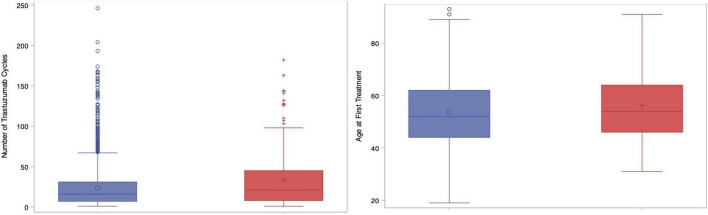
Box plots showing number of trastuzumab cycles (left) and age at first treatment (right) compared between groups. Blue = Group A; Red = Group B.

For the primary endpoint, the incidence of CEs was 7.3% (*n* = 167) in what will herein be referred to as group A, whereas 92.3% (*n* = 2117) did not have any CEs (group B). Table 1 contains baseline characteristics for both the study population overall as well as each study group individually. Heart failure was observed in 6.9% (*n* = 158) of the study population, representing 94.6% (158/167) of the CEs. Less than 1% of patients died of cardiac causes (*n* = 12, 0.5%) while 34.1% (*n* = 779) died of their cancer, and 14.8% died for other reasons (*n* = 339). Group A had an older median age of 61 (IQR 51–70) vs. 57 (48–66) in group B, *p* < 0.01. Patients in group A also received a greater number of trastuzumab cycles, 21.0 (IQR 8–45) vs. 16.0 (IQR 7–31), *p* < 0.01. Figure 1 contains boxplots showing the distribution of age and trastuzumab cycles in groups A and B. The remaining baseline characteristics were well-balanced between groups A and B with no significant differences found for neighborhood income quintiles, pertuzumab use, prior anthracycline use, prior trastuzumab use, cancer related deaths, baseline Charlson co-morbidity index, estrogen receptor status, progesterone receptor status, or initial stage at diagnosis (I-III vs. IV). In total, the Charlson index was 0 in 28.2% (*n* = 644), 1–2 in 10.7% (*n* = 244) and ≥3 in 50.9% (1,163).

**TABLE 1:** Baseline characteristics.

Variable	Value	Overall study population	Group A: incidence of cardiac event	Group B: no cardiac event	*P*-value
	
	N	2,284	167	2,117	
Year of treatment*	2007	202 (8.8%)	11 (6.6%)	191 (9.0%)	0.004
	2008	221 (9.7%)	24 (14.4%)	197 (9.3%)	
	2009	189 (8.3%)	19 (11.4%)	170 (8.0%)	
	2010	174 (7.6%)	15 (9.0%)	159 (7.5%)	
	2011	211 (9.2%)	17 (10.2%)	194 (9.2%)	
	2012	235 (10.3%)	16 (9.6%)	219 (10.3%)	
	2013	252 (11.0%)	27 (16.2%)	225 (10.6%)	
	2014	231 (10.1%)	16 (9.6%)	215 (10.2%)	
	2015	245 (10.7%)	14 (8.4%)	231 (10.9%)	
	2016	220 (9.6%)	8 (4.8%)	212 (10.0%)	
	2017	104 (4.6%)	0 (0.0%)	104 (4.9%)	
Age at first treatment	Mean ± SD	57.67 ± 12.94	61.10 ± 12.54	57.40 ± 12.94	<0.001
	Median (IQR)	57.00 (49.00–66.00)	61.00 (51.00–70.00)	57.00 (48.00–66.00)	0.001
	<40	197(8.6%)	≤5 (2.4%)	193(9.1%)	0.008
	40–49	491 (21.5%)	35 (21.0%)	456 (21.5%)	
	50–59	691 (30.3%)	44 (26.3%)	647 (30.6%)	
	60–69	517 (22.6%)	45 (26.9%)	472 (22.3%)	
	70–79	269 (11.8%)	25 (15.0%)	244 (11.5%)	
	≥80	119(5.2%)	14 (8.4%)	105 (5.0%)	
Charlson co-morbidity index	0	644 (28.2%)	57 (34.1%)	587 (27.7%)	0.317
	1–2	244 (10.7%)	18 (10.8%)	226 (10.7%)	
	≥3	1,163 (50.9%)	75 (44.9%)	1,088 (51.4%)	
	missing	233 (10.2%)	17 (10.2%)	216 (10.2%)	
ER status	Negative	444 (19.4%)	30 (18.0%)	414 (19.6%)	0.611
	Positive	556 (24.3%)	37 (22.2%)	519 (24.5%)	
	Missing	1,284 (56.2%)	100 (59.9%)	1,184 (55.9%)	
PR status	Negative	566 (24.8%)	40 (24.0%)	526 (24.8%)	0.538
	Positive	434 (19.0%)	27 (16.2%)	407 (19.2%)	
	Missing	1,284 (56.2%)	100 (59.9%)	1,184 (55.9%)	
Stage at diagnosis	Previous stage I-III	854 (37.4%)	61 (36.5%)	793 (37.5%)	0.811
	Stage IV	1,430 (62.6%)	106 (63.5%)	1,324 (62.5%)	
Trastuzumab in previous 5 years	No	2,166 (94.8%)	154 (92.2%)	2,012 (95.0%)	0.112
	Yes	118 (5.2%)	13 (7.8%)	105 (5.0%)	
Concurrent pertuzumab	No	1,712 (75.0%)	134 (80.2%)	1,578 (74.5%)	0.102
	Yes	572 (25.0%)	33 (19.8%)	539 (25.5%)	
Received anthracyclines in previous 10 years	No	1,692 (74.1%)	120 (71.9%)	1,572 (74.3%)	0.496
	Yes	592 (25.9%)	47 (28.1%)	545 (25.7%)	
Received anthracyclines in previous 5 years	No	1,762 (77.1%)	126 (75.4%)	1,636 (77.3%)	0.588
	Yes	522 (22.9%)	41 (24.6%)	481 (22.7%)	
Heart failure during treatment	No	2,126 (93.1%)	9 (5.4%)	2,117 (100.0%)	<0.001
	Yes	158 (6.9%)	158 (94.6%)	0 (0.0%)	
Deceased	No	1,154 (50.5%)	78 (46.7%)	1,076 (50.8%)	0.305
	Yes	1,130 (49.5%)	89 (53.3%)	1,041 (49.2%)	
Cause of death: breast cancer	No	1,505 (65.9%)	117 (70.1%)	1,388 (65.6%)	0.238
	Yes	779 (34.1%)	50 (29.9%)	729 (34.4%)	
Cause of death: cardiac	No	2,272 (99.5%)	155 (92.8%)	2,117 (100.0%)	<0.001
	Yes	12 (0.5%)	12 (7.2%)	0 (0.0%)	
Cardiac tests/year	Mean ± SD	2.26 ± 1.67	3.22 ± 2.17	2.18 ± 1.60	<0.001
	Median (IQR)	2.00 (1.00–3.00)	3.00 (2.00–5.00)	2.00 (1.00–3.00)	<0.001
Any cardiac testing	Cardiac testing	1,964 (86.0%)	154 (92.2%)	1,810 (85.5%)	0.016
	No cardiac testing	320 (14.0%)	13 (7.8%)	307 (14.5%)	
Total number of cardiac tests	Mean ± SD	4.54 ± 5.33	7.78 ± 7.71	4.29 ± 5.01	<0.001
	Median (IQR)	3.00 (1.00–6.00)	5.00 (2.00–11.00)	3.00 (1.00–6.00)	<0.001
Number of cardiologist visits	One or more cardiac visits	383 (16.8%)	110 (65.9%)	273 (12.9%)	<0.001
	No cardiac visits	1,901 (83.2%)	57 (34.1%)	1,844 (87.1%)	
Hospital type	Community	1,248 (54.6%)	84 (50.3%)	1,164 (55.0%)	0.242
	Teaching	1,036 (45.4%)	83 (49.7%)	953 (45.0%)	
Neighborhood income quintile	Lowest income, Q1	395 (17.3%)	24 (14.4%)	371 (17.5%)	0.357
	Q2	446 (19.5%)	31 (18.6%)	415 (19.6%)	
	Q3	435 (19.0%)	27 (16.2%)	408 (19.3%)	
	Q4	508 (22.2%)	47 (28.1%)	461 (21.8%)	
	Highest income, Q5	490 (21.5%)	38 (22.8%)	452 (21.4%)	
	Missing	10 (0.4%)	0 (0.0%)	10 (0.5%)	
Number of cycles trastuzumab	Mean ± SD	24.82 ± 27.66	33.81 ± 36.21	24.11 ± 26.75	<0.001
	Median (IQR)	16.00 (7.00–32.00)	21.00 (8.00–45.00)	16.00 (7.00–31.00)	0.001

*Each year provides a percentage of the total study population.

### Risk Factors for Cardiac Events

**FIGURE 2:**
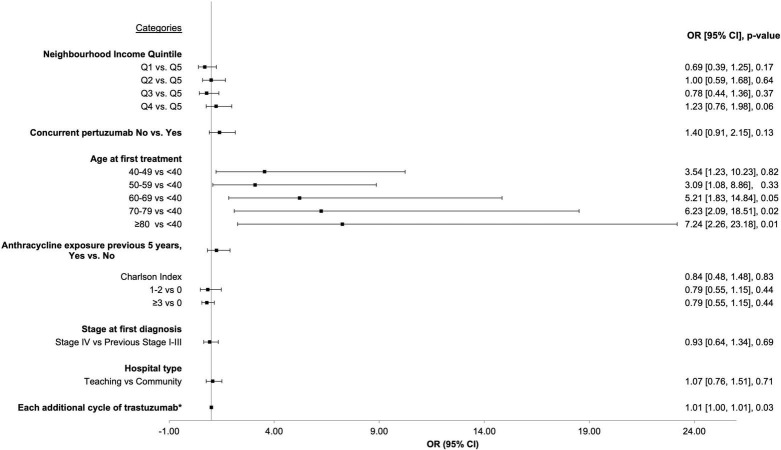
Forrest plot depicting results from logistic regression for factors associated with increased odds of cardiac event.

Logistic regression was performed to determine if there were clinical factors that increased the odds of developing CEs during or immediately after trastuzumab. Age, neighborhood income quintile, concurrent pertuzumab use, Charlson co-morbidity index, prior anthracycline use, recurrent or *de novo* metastatic disease, hospital setting (teaching vs. community), and number of cycles of trastuzumab were included in the model. Older age (analyzed in 10-year blocks) vs. age < 40 was the most significant factor increasing the odds of CE: age 60–69, OR = 5.21 (95% CI 1.83–14.84, *p* = 0.05); age 70–79, OR = 6.23 (95% CI 2.09–18.51, *p* = 0.02); age > 80, OR = 7.24 (95% CI 2.26–23.18, *p* = 0.01). When treated as a continuous variable, each additional cycle of trastuzumab lead to a statistically significant increase in the odds of a CE (OR 1.01, 95% CI 1.00–1.01, *p* = 0.03). Figure 2 depicts the Forrest Plot with the complete results of logistic regression presented in Table 2.

**TABLE 2:** Results from logistic regression showing factors associated with incidence of cardiac events in advanced HER2 + breast cancer patients, 2007–2017.

Categories	OR (95% CI)	*p*-Values
**Neighborhood income quintile**		
Q1 vs. Q5	0.69 (0.39–1.25)	0.17
Q2 vs. Q5	1 (0.59–1.68)	0.64
Q3 vs. Q5	0.78 (0.44–1.36)	0.37
Q4 vs. Q5	1.23 (0.76–1.98)	0.06
Concurrent pertuzumab no vs. yes	1.4 (0.91–2.15)	0.13
**Age at first treatment**		
40–49 vs. <40	3.54 (1.23–10.23)	0.82
50–59 vs. <40	3.09 (1.08–8.86)	0.33
60–69 vs. <40	5.21 (1.83–14.84)	0.05
70–79 vs. <40	6.23 (2.09–18.51)	0.02
≥80 vs. <40	7.24 (2.26–23.18)	0.01
Anthracycline exposure previous 5 years, yes vs. no	1.25 (0.82–1.9)	0.31
**Charlson index**		
1–2 vs. 0	0.84 (0.48–1.48)	0.83
≥3 vs. 0	0.79 (0.55–1.15)	0.44
**Stage at first diagnosis**		
Stage IV vs. previous stage I–III	0.93 (0.64–1.34)	0.69
**Hospital type**		
Teaching vs. community	1.07 (0.76–1.51)	0.71
Each additional cycle of trastuzumab*	1.01 (1–1.01)	0.03

*Cycles of trastuzumab treated as continuous variable.

### Cardiac Monitoring

The final outcome of interest was to understand cardiac monitoring patterns in HER2 + aBC. The overall study population had a median of 2 (IQR 1–3) cardiac tests per year. Patients with CEs had more cardiac imaging (*p* < 0.01) and a median three tests per year (IQR 2–5) vs. 2 (IQR 1–3) in those with no CEs. Overall, patients in group A had a median of 5 cardiac tests (2–11) vs. 3 (1–6) in group B, *p* < 0.01. Group A was also more likely to have seen a cardiologist during the time they were on trastuzumab 65.9 vs. 12.9% (*p* < 0.01). Of note, 14% (*n* = 307) of patients had no captured cardiac imaging at all, 7.8% (*n* = 13) in this group had CEs.

## Discussion

In patients with HER2 + breast cancer, anti-HER2 targeted therapy has dramatically improved cancer outcomes. Trastuzumab-based therapy is associated with an increased risk of cardiotoxicity in patients with early-stage disease with most cases observed within 18 months of completing treatment. There is a paucity of information on cardiotoxicity of anti-HER2 targeted agents in patients with aBC. Our study is the largest to report cardiac outcomes in aBC treated with trastuzumab and/or pertuzumab in a non-clinical trial setting. In this retrospective population-based study of HER2 + aBC, the incidence of cardiac events during or after trastuzumab-based therapy was 7.3%, (*n* = 167) with heart failure representing the majority of these events (6.9%, *n* = 158). These findings are similar to the 3–7% reported in the literature (6, 8, 14), but lower than a Dutch study of 429 patients likely given the reported combined incidence of severe and non-severe cardiotoxicity based on LVEF parameters of 11.7% and 9.1% after 1 and 2 years of trastuzumab respectively (21); after 4 years of treatment, the cumulative incidence of severe cardiotoxicity (LVEF < 40%) was 3.1%.

Although our study demonstrates a higher incidence of CEs in the community than reported in prior clinical trial studies (7.3 vs. 1–2%), there were very few deaths from cardiac causes (1%) in our study population while more than a third of all deaths were attributable to breast cancer (69%). During the timeframe of our data collection period, 49.4% of patients were still alive which is consistent with a near 5-year median overall survival seen in this population (16). These are important results to consider when weighing the risks and benefits of treatment for aBC. The risk of cardiotoxicity related to anti-HER2 targeted therapy rarely translates into a greater mortality risk to the patient then their cancer. However, holding or delaying these agents precludes patients from life-prolonging therapy. In our study population, there were no differences in the incidence of CEs in patients with routine cardiac imaging as opposed to those with no cardiac imaging. Therefore, clinicians may decide that only patients with the presence of reliable predictors of CEs should undergo interval cardiac evaluations. In our cohort, older age and longer duration of trastuzumab-based therapy were predictive of CEs however given the borderline statistical significance (HR = 1.01) these results should be interpreted with caution when considering frequency of cardiac monitoring. Our results are consistent with findings from the Dutch group which observed that older age and a baseline reduced LVEF increased the risk of severe cardiotoxicity (21).

The results of our study challenges the practice of routine cardiac imaging in aBC patients treated with anti-HER2 targeted therapy (9–11) as per the current FDA and Cancer Care Ontario recommendations. In Ontario, the current cost of an echocardiogram is $2000 CAD (22). This would translate to more than $6,000 CAD in echocardiogram costs per individual as per a cohort-based analysis in the United States where patients treated with trastuzumab in the aBC setting had an average of 3.1 echocardiograms over the course of their treatment (23).

In our study, cardiac imaging was not available in a minority of patients (*n* = 307); these patients experienced a similar CE rate (7.8%, *n* = 13) compared to those patients with regular cardiac monitoring (group A: CE = 7.3%, *n* = 167). Future guidelines on cardiac monitoring in HER2 + aBC should consider the clinical benefit of anti-HER2 therapy in the context of the low incidence of CEs. Targeted LVEF monitoring should focus on those considered to be at higher risk of developing cardiotoxicity; patients over 60 or those who are treated for more than 1 year with anti-HER2 targeted therapy, while all other patients should be monitored clinically for cardiotoxicity. This approach would reduce the number of cardiac investigations and focus clinical decision making on quality of life and efficacy of anti-HER2 treatment. Another potential method suggested by the European Society of Medical Oncology is monitoring of cardiac biomarkers such as NT-pro-BNP and troponin (9). NT-proBNP is a known prognostic and predictive biomarker in heart failure (24). Although its predictive use in the context of patients treated with cardiotoxic cancer therapy has been inconsistent, a recent large prospective study of 323 patients observed consistent associations between increased in NT-proBNP and LVEF declines particularly notable in the sequential anthracycline and trastuzumab group (25). Therefore, the use of a dynamic risk prediction model combining clinical risk factors and serial biomarkers should be an avenue of future research. Population-based registries provide valuable information on the incidence of cardiotoxicity in patients with HER2 + aBC exposed to multiple lines of anti-HER2 targeted therapy, facilitating the development of risk stratification models to determine the optimal cardiac imaging strategy for individual patients.

There are some limitations to our study. Firstly, it is limited by its nature of being a retrospective database review. While the source of data from ICES is rich, it is only as strong as the data entered into the system. Within the provincial database, we did not have access to patient level data including LVEF. Furthermore, the database does not permit access to patients prescribed oral medications or details about participation in clinical trials. We were also not able to clearly discern which line of therapy patients received trastuzumab due to differences in recording this data. There is a possibility of misclassification of the outcome in our study due to the outcome definition being based on diagnostic codes and hospital visits. Using this approach, we likely overestimate the incidence of severe or clinically significant cardiotoxicity in our cohort as it is conceivable that patients were referred to cardiology for asymptomatic changes in left ventricular function. The administrative data used in this study may not capture care that is continued and provided at other hospitals. Furthermore, our study excluded patients with a prior history of heart failure and therefore we were not able to assess the risk associated with that comorbidity. We also acknowledge that the proportion of *de novo* stage IV cancer patients was higher than expected which may reflect timing and limitations within the database. In comparison, the MA.31 trial also had a large portion of patients (43%) with *de novo* stage IV HER2 + disease. This however is unlikely to impact our primary endpoints. Given that this is a population-based study which relies on reporting, there is a selection bias toward patients with clinical heart failure and although recent guidelines recommend baseline imaging, these were not standard of care during the chosen study period. In terms of therapy, this study was limited to trastuzumab and pertuzumab and did not include other anti-HER2 targeted therapies (e.g., lapatinib, neratinib) used in the advanced setting which limits its generalizability. Finally, we did not capture treatments received subsequent to trastuzumab and thus cannot comment on the impact of those agents.

In conclusion, this is the largest population-based study to report CEs in aBC patients treated with trastuzumab-based therapy. The incidence of CEs was low and cardiac death was very rare. More than a third of deaths were attributable to breast cancer. Routine cardiac imaging was performed per provincial guidelines in most patients, but the rates of cardiac events were comparable regardless of whether or not patients had surveillance LVEF assessments. Our results combined with other recent cohort studies challenge the requirement for routine cardiac monitoring in this patient population. Given the chronicity of this disease, patients may be on anti-HER2 targeted therapy for several years with exposure to serial cardiac monitoring with little clinical benefit. We recommend using a personalized risk-based approach that incorporates clinical risk factors and cardiovascular health. Further research on defining the best cardiac imaging techniques with incorporation of cardiac biomarkers is needed to optimize cardiac surveillance strategies in this patient population.

## Data Availability Statement

The raw data supporting the conclusions of this article will be made available by the authors, without undue reservation.

## Ethics Statement

This study was reviewed and approved by the Ottawa Health Sciences Research Ethics Board, Ottawa, Canada.

## Author Contributions

MR contributed to the analysis and interpretation of the results, the design of the tables and figures, and writing of the manuscript. CK contributed to the design of the tables and figures and writing of the manuscript. SD contributed to organization and supervision of the research. MR, CK, IL, MT, KP, SH, and SD contributed to the literature review, writing of the manuscript, and critical review. All authors contributed to the article and approved the submitted version.

## Author Disclaimer

The opinions, results, and conclusion reported in this manuscript are those of the authors and are independent from the funding sources. No endorsement by ICES or the Ontario MOHLTC is intended or should be inferred. Parts of this material are based on data and/or information compiled and provided by the Canadian Institute for Health Information (CIHI). However, the analyses, conclusion, opinions, and statements expressed in the material are those of the author(s), and not necessarily those of CIHI.

## Conflict of Interest

The authors declare that the research was conducted in the absence of any commercial or financial relationships that could be construed as a potential conflict of interest.

## Publisher’s Note

All claims expressed in this article are solely those of the authors and do not necessarily represent those of their affiliated organizations, or those of the publisher, the editors and the reviewers. Any product that may be evaluated in this article, or claim that may be made by its manufacturer, is not guaranteed or endorsed by the publisher.
